# Gametophyte and embryonic ontogeny: understanding the reproductive calendar of *Cypripedium japonicum* Thunb. (Cypripedoideae, Orchidaceae), a lady’s slipper orchid endemic to East Asia

**DOI:** 10.1186/s12870-020-02589-9

**Published:** 2020-09-15

**Authors:** Balkrishna Ghimire, Sungwon Son, Jae Hyeun Kim, Mi Jin Jeong

**Affiliations:** 1 Division of Forest Biodiversity, Korea National Arboretum, Pocheon, 11186 South Korea; 2 Division of Plant Resources, Korea National Arboretum, Yongmun, 12519 South Korea

**Keywords:** *Cypripedium japonicum*, Gametophyte development, Embryology, Reproductive calendar, Endangered species

## Abstract

**Background:**

The genus *Cypripedium* L. is one of the five genera of the subfamily Cypripedioideae, members of which are commonly known as lady’s slipper orchids. *Cypripedium japonicum* is a perennial herb native to East Asia, specifically China, Japan, and Korea. Due to its limited distribution, the species is included in the Endangered category of the IUCN Red List.

**Results:**

We investigated gametophyte development, including complete embryogenesis, in *C. japonicum*. The complete reproductive cycle is presented based on our observations. Anther development begins under the soil, and meiosis of pollen mother cells begins 3 weeks before anthesis, possibly during early April. The megaspore mother cells develop just after pollination in early May and mature in mid–late June. The pattern of embryo sac formation is bisporic, and there are six nuclei: three forming the egg apparatus, two polar nuclei, and an antipodal cell in the mature embryo sac. Triple fertilization results in the endosperm nucleus, which degenerates when the proembryo reaches the eight-to-sixteen-cell stage.

**Conclusion:**

Our overall comparisons of the features of gametophyte and embryo development in *C. japonicum* suggest that previous reports on the embryology of *Cypripedium* are not sufficient for characterization of the entire genus. Based on the available information, a reproductive calendar showing the key reproductive events leading to embryo formation has been prepared.

## Background

The family Orchidaceae is one of the largest families of flowering plants, along with Asteraceae [1]. The orchid taxa exhibit an unusual pattern of embryo development, and the diverse morphology of the suspensor is one of the most distinctive features of the whole family [1, 2]. The characteristic suspensor development, patterns of polyembryony, and vast diversity of megagametophyte development in orchids provide continual fascination for plant biologists investigating the embryology of this family [2–5]. The extraordinarily diverse pattern of suspensor morphology in orchids motivated Swamy [4] to conceive a classification scheme for embryo development in Orchidaceae. In addition, anthers usually develop much earlier than ovules in orchid species; at pollination, when anthers release mature pollen grains containing fully developed vegetative and generative cells, the ovule has yet to produce megaspore mother cells (MMCs [6, 7]). It has been suggested that ovule development is activated by pollination, after which the plant takes several weeks to produce a female gamete [8]. Zhang and O’Neill [9] believed that the commonly observed prolonged periods of ovule development in the family makes Orchidaceae an intriguing subject for embryological study.

The genus *Cypripedium* L. is one of the five genera of the subfamily Cypripedioideae, commonly known as lady’s slipper [1, 10]. Habitat destruction and overcollection have demographic implications, leading to the decline and regional extinction of populations of *Cypripedium* spp. [11–13]. On the other hand, low conversion rates of flowers to fruit remain a major issue in the reproductive ecology of existing populations of *Cypripedium* spp. [14]. All the species of this group have a pouch-like lip, two fertile stamens, a shield-like staminode, and a synsepal with fused lateral sepals, and this specific flower morphology does not offer an easy reward to pollinators. A unique feature of Cypripedioideae orchids is the one-way trap flower, which offers a fixed and unidirectional route to pollinators (see [15]). Despite this fixed pollination route, slipper orchids exhibit a remarkable degree of diversity in food mimesis traits such as scent, color and flower size for their pollinators [15–19].

The genus *Cypripedium* comprises approximately 45–50 species that are mostly found in temperate zones of the Northern Hemisphere, mainly in temperate regions of Asia and North America, extending to the Himalayan regions and Central America [1, 10, 20]. The center of diversity of the genus lies in eastern Asia, where 38 species have been reported; in particular, China alone has an astonishing diversity of this genus, harboring 36 species of which 25 species are endemic [20]. Many species of *Cypripedium* in North America and Europe have become rare due to overexploitation and illegal trade in past centuries [12, 21]. The status of *Cypripedium* in northeastern Asia is no different. Twenty-four Asian *Cypripedium* species are currently included in the Endangered or Critically Endangered category of the IUCN Red List [22]. In addition, the Convention on International Trade in Endangered Species of Wild Fauna and Flora (CITES) currently lists all *Cypripedium* species in CITES Appendix II [23].

*Cypripedium japonicum* Thunb. is an endemic species of the endangered East Asian lady slipper orchid distributed across China, Japan, and Korea. It grows in damp and humus-rich soil in forests or on shady slopes along ravines at an altitude of 1000–2000 m. Due to its limited distribution and small populations in its natural habitat, this species has been categorized as critically endangered at the national level in Korea (although it is endangered globally) [24]. Correspondingly, in China and Japan, the species is at a high risk of extinction due to habitat loss and anthropogenic activities such as overcollection for horticultural and medicinal purposes [25, 26].

Empirical knowledge of plant reproductive biology, particularly for rare orchids, could help us determine whether inadequacy in the recruitment cycle controls successful reproduction and constrains population growth [27]. A few recent efforts have been made to study the pollination biology and reproductive characteristics of *C. japonicum* [28, 29]. In the early twentieth century, Pace [30] wrote a comprehensive account of fertilization in the genus *Cypripedium,* although *C. japonicum* was not examined. Later, Sood and Rao [31] studied the embryology of *Cypripedium cordigerum* and confirmed that the embryological characteristics and pericarp structure in this species were typical orchidaceous types. Although a majority of orchids follow a monosporic pattern of embryo sac development [32, 33], a bisporic pattern has been described for *Cypripedium* and *Paphiopedilum* [34, 35]. While there was no structural or developmental examination of antipodal cells in Pace’s [30] report, Poddubnaya-Arnoldi [36] described the well-developed antipodal cells that undergo secondary multiplication in some *Cypripedium* species. In addition, most orchids fail to form a functional endosperm, although some degree of endosperm development with a multinucleate stage in some species has been reported [30, 31, 36]. This failure to form a triploid endosperm is one of the unique features in orchid seed development. In recent years, several studies have been carried out focusing on the embryology of different orchid species [2, 37–40]. However, little is known about the embryology and reproductive biology of *C. japonicum*. Knowledge of gametophyte, embryo, and seed development is essential for understanding successful reproduction in this endangered orchid and for formulating a restoration strategy.

We investigated the detailed embryology and seed formation processes in this popular lady’s slipper orchid. The primary objectives of this study were (1) to investigate male and female gametophyte development in artificially pollinated *C. japonicum*; (2) to understand the fertilization, embryo, and seed development processes in *C. japonicum*; and (3) to develop a complete reproductive calendar of the species showing key reproductive events leading to seed development.

## Results

### Microsporogenesis and microgametogenesis

**Fig. 1:**
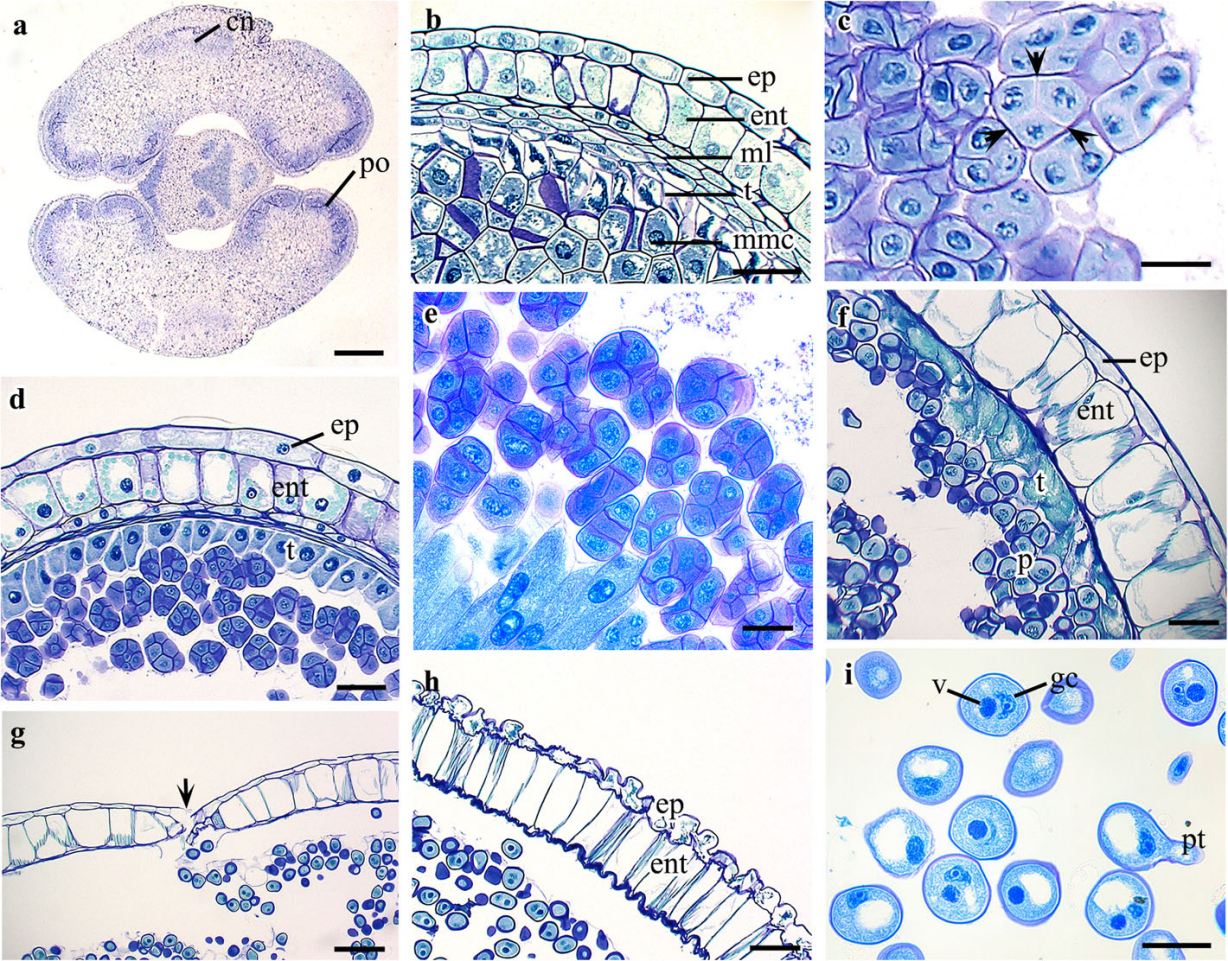
Development of anther and pollen grain in *Cypripedium japonicum*. **a**. Cross section (CS) of young pollinia. **b**. CS of young pollinia showing wall layers. **c**. Cytokinesis in microspores. **d**. CS of pollinia showing anther wall and microspore tetrads. **e**. CS of mature pollinia with variously shaped pollen tetrad/polyads. **f**, **g**. CS of mature pollinia showing development of wall thickenings in endothecium and dehiscence of anther. **h**. Mature anther wall with papillate epidermis and fibrous thickenings in endothecium. **i**. Two nucleate mature pollen grains. *Abbreviations*: ent, endothecium; ep, epidermis; gc, generative cell; ml, middle layer; mmc, microspore mother cell; po, pollinia; pt., pollen tube; t, tapetum; v, nucleus of the vegetative cell. Scale bars: **a** = 500 μm, **b**, **d** = 50 μm, **c-i** = 30 μm, **e**, **f**, **g**, **h**= 100 μm

The anther is bilobed and tetrasporangiate (Fig. 1a). Prior to maturation, the anther wall comprises five or six layers: an epidermis, an endothecium, two or three middle layers, and a tapetum (Fig. 1b). Unfortunately, due to a lack of young anthers, we could not confirm the process of anther wall development. The tapetum is glandular, and its cells are uninucleate throughout the developmental process (Fig. 1b, d). The middle layers degenerate at the same time that the spore tetrads/polyads give rise to free spores (Fig. 1f). Meanwhile, radial expansion is followed by the development of fibrous thickening in the endothecium cells, and individual cells become highly vacuolated (Fig. 1f). At the same time, the nuclei of the endothecium cells start to degenerate, and this process is completed when pollen grains reach the binucleate stage (Fig. 1g, h). As the anther expands, the epidermal cells elongate tangentially, and their nuclei degenerate, either before or after spore formation. Both the epidermis and endothecium are persistent (Fig. 1g, h). In mature anthers, the epidermis is papillate and becomes shiny and sticky on the outer surface with dark brown edges during anthesis (field observation).

The sporogenous cells form an arc-like structure in the anther lobe, which differentiates into microspore mother cells (Fig. 1a, b). The microspore mother cells undergo meiotic division, resulting in microspores. Cytokinesis in the microspore tetrad is always simultaneous, and the resultant tetrad is tetrahedral, isobilateral, T-shaped, or linear (Fig. 1c, d, e). Unequal mitotic division occurs in the microspore, resulting in a smaller ellipsoidal generative cell and a larger vegetative cell with a rounded nucleus; thus, pollen grains are binucleate during anthesis (Fig. 1h, i). It is possible that no further division occurs before pollination, although some pollen grains develop a pollen tube inside the anther (Fig. 1i). Anther dehiscence takes place from a common longitudinal slit between two chambers of the same lobe (Fig. 1g).

### Ovule

**Fig. 2:**
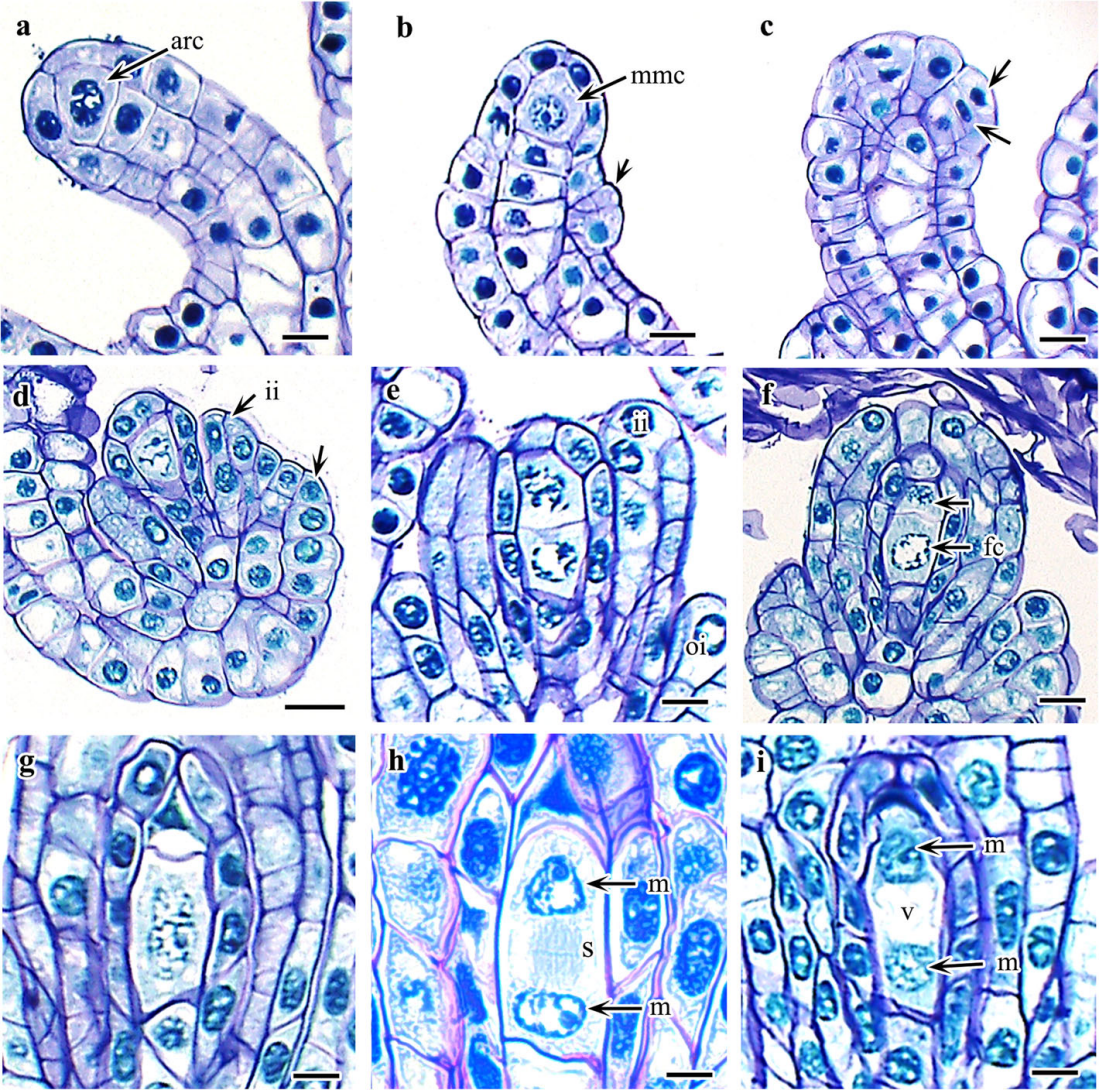
Development of ovule and megagametophyte in *Cypripedium japonicum*. **a**. Young ovular filament with archesporial cell. **b**-**c**. Young ovule with megaspore mother cell (MMC) and initiation of integument (arrow in C indicate plane of division in dermal cell). **d**. Anatropous, bitegmic ovule. **e**. First phase of meiosis division in MMC. **f**. Two unequal megaspore dyad (arrow indicates degenerating dyad of megaspore). **g**. Elongated functional dyad of megaspore. **h**–**i**. Meiosis II in functional dyad of megaspore. *Abbreviation*: arc, archesporial cell; fc, functional dyad of megaspore; ii, inner integument; m, nucleus of functional dyad of megaspore; mmc, megaspore mother cells; oi, outer integument; s, spindle; v, vacuole. Scale bars:  **a**= 500 μm, **b**, **c**, **e-i**= 20 μm,  **d**= 50 μm

Each of the branched nucellar filaments raised in the placental ridge of the ovary comprises approximately four to seven nucellar cells surrounded by a single layer of nucellar epidermis (Fig. 2a). The topmost nucellar cell becomes enlarged and functions as an archesporial cell. Meanwhile, integuments start to initiate in the nucellar filament. The origin of the integuments in the ovule primordium is dermal (Fig. 2b, c). The inner integument differentiates first, becomes two-cell-layered, and eventually forms the micropyle before fertilization. The outer integument differentiates slightly later but in a similar fashion as the inner integument and grows beyond the inner integument after fertilization. During this period, nucellar filaments regularly proliferate and begin to bend back toward the placental ridge and finally exhibit an anatropous condition. The mature ovule is therefore tenuinucellate, bitegmic, and anatropous in nature (Fig. 2b, d).

### Megasporogenesis and megagametogenesis

**Fig. 3:**
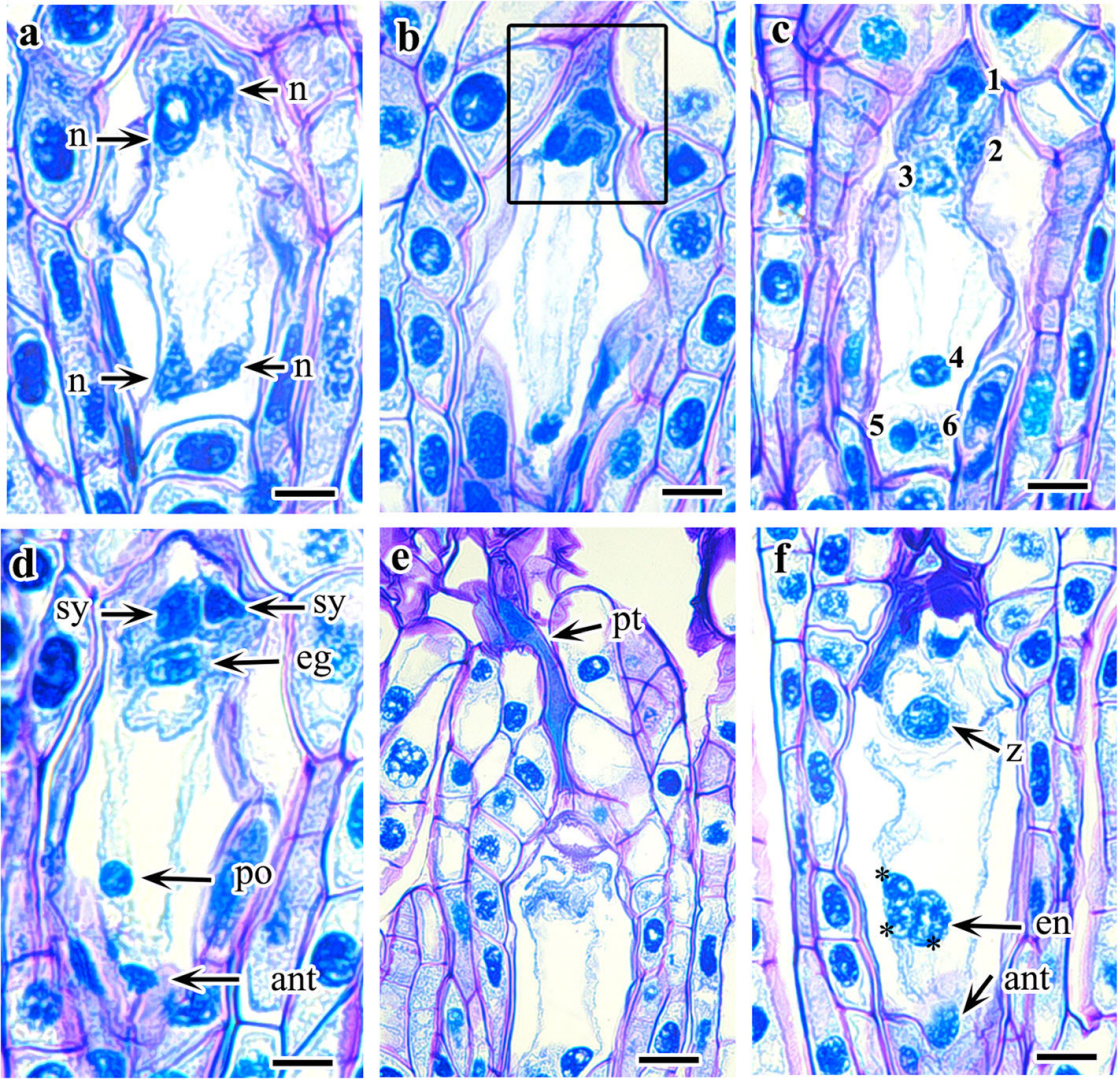
Development megagametophyte and zygote in *Cypripedium japonicum*. **a**. Four nucleate embryo sac formed by first mitotic division in nucleus of functional dyad. **b**. Micropylar nuclei after second mitotic division (rectangle line). **c**-**d**. Six nucleate mature embryo sac. **e**. Pollen tube. **f**. Zygote and endosperm nucleus (asterisks in central cell indicate triple fusion). *Abbreviations:* ant, antipodal cell; eg, egg cell; en, endosperm nuclei; n, megaspore nucleus; po, polar nuclei; pt., pollen tube; sy, synergid; z, zygote. Scale bars:  **a-h**= 20 μm

The single archesporial cell increases in size and directly functions as an MMC (Fig. 2b). Further enlargement of the MMC is followed by the first phase of meiotic division to form two dyad cells, of which only the chalazal cell is functional (Fig. 2d–f). The micropylar cell of the dyad soon degenerates, probably before or just after the second meiotic division, and the entire embryo sac development occurs from the chalazal cell of the dyad. Together with degeneration of the micropylar cell, the functional chalazal cell of the dyad becomes enlarged and elongated (Fig. 2g). The second meiotic division in the chalazal cell of the dyad produces two primary megaspore nuclei (Fig. 2h). The second meiotic division is immediately followed by shifting of the nuclei to opposite poles of the cell (Fig. 2i). A large vacuole is formed during the endosporial germination of a functional megaspore, which results in the shifting of the nuclei to the poles. The first mitotic division in the primary micropylar and chalazal nuclei occurs after complete shifting of the nuclei to the opposite pole, resulting in the formation of a four-nucleate embryo sac with two nuclei at each pole (Fig. 3a). At this stage, two micropylar nuclei undergo second mitotic division to form four nuclei, but no further division is observed in the two chalazal nuclei (Fig. 3b). Thus, the mature embryo sac contains six nuclei (Fig. 3c). The four micropylar nuclei form an egg cell, two synergids, and one polar nucleus, whereas the two chalazal nuclei form the polar nucleus and an antipodal cell (Fig. 3d).

### Pollination and fertilization

The anther matures much earlier than the ovule. The ripened anther is yellow with a shiny, sticky surface on the outer wall of the epidermis. The flowers were hand-pollinated from May 2–5. The sticky surface of the anther helps attach the anther to the stigma. During pollination, pollen grains are binucleate, and the pollen tube starts to develop. In contrast, the nucellar filaments are either just ready or still not ready to organize the archesporial cells. The generative nucleus possibly divides inside the pollen tube, forming two male gametes. After pollination, the pollen tubes grow along the style. We were not able to observe pollen tube growth in the stylar canal. However, a spreading mass of pollen tubes on placental lobes was observed at 21 days after pollination (DAP) during meiosis on MMCs near completion, and the embryo sac entered two nuclear stages. An entangled mass of pollen tubes remains near the placenta cells until megasporogenesis is complete. Fertilization occurs almost 7–8 weeks after pollination (WAP). Fertilization is porogamous: the pollen tube enters the ovule through the micropyle, resulting in a diploid embryonic zygote by fusion of one male gamete with an egg and a triploid endosperm zygote after the fusion of one male gamete with a diploid polar nucleus (Fig. 3e, f). The single antipodal cell is ephemeral in nature and degenerates soon after fertilization (Fig. 3f). The endosperm zygote divides once and becomes binucleate; however, it does not develop further and degenerates before the embryo occupies the whole space.

### Embryogenesis

**Fig. 4:**
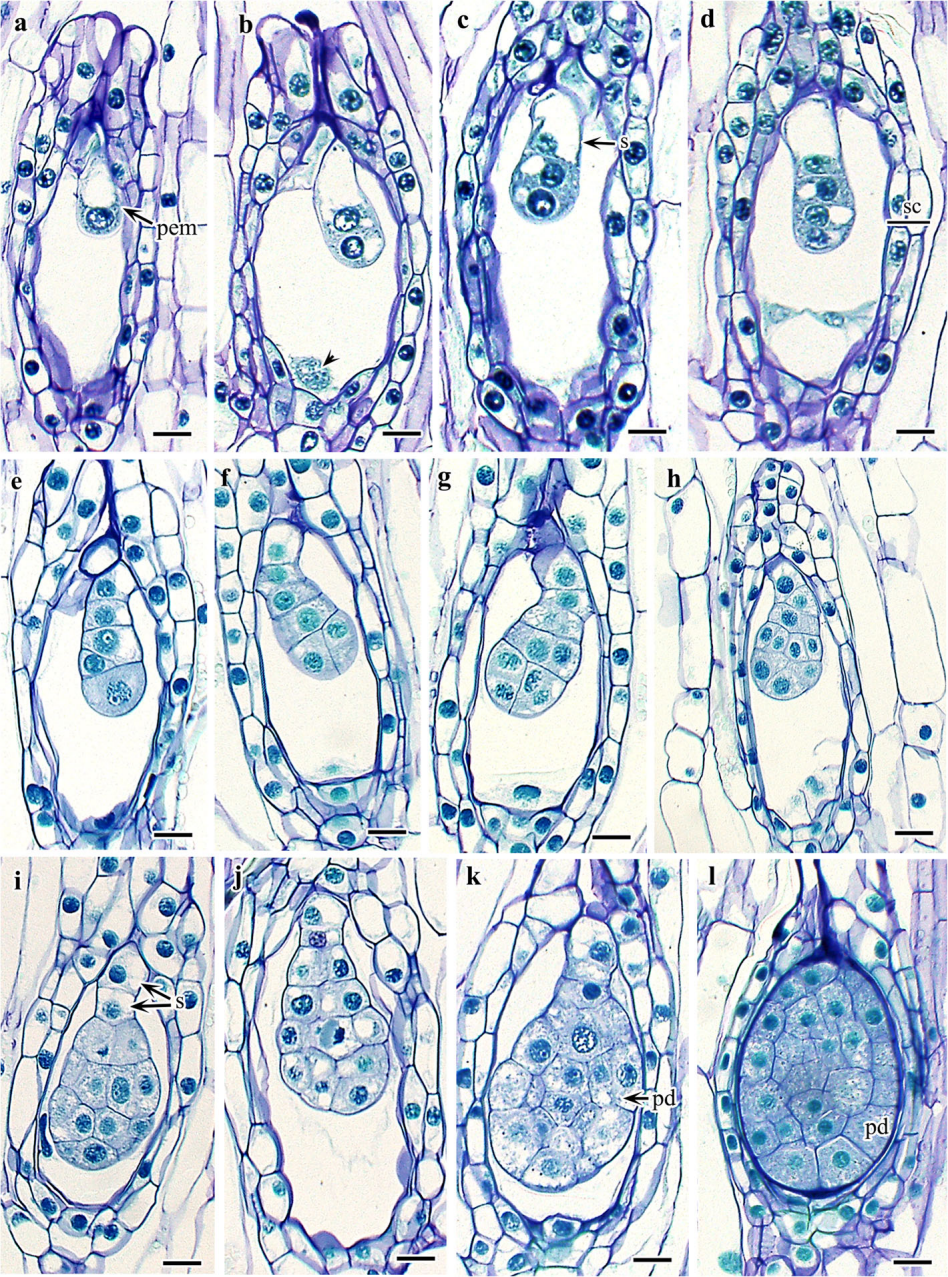
Development of embryo in *Cypripedium japonicum*. **a**. Developing pro-embryo. **b**. First division in pro-embryonal cell followed by elongation of micropylar cell forming unequal size of two embryonal cells. **c**. Second transverse division in chalazal cell forming three celled pro-embryo (two chalazal, and a micropylar). **d**. Third division in chalazal cell forming four-celled linear pro-embryo. **e**. Five celled linear pro-embryo (micropylar cell divides in this stage forming two celled suspensor). **f**. Longitudinal division in chalazal cell of five celled embryo showing two juxtaposed cells. **g**–**i**. Pro-embryo forming early stage of globular pro-embryo (endosperm nucleus completely degenerates after this stage). **j**–**k**. Periclinal division in embryonic cell (except two basal suspensor cells) forming protoderm layer of embryo. **l**. Fully developed embryo covering whole inner space of the seed. *Abbreviations*: pem, pro-embryo pd., protoderm layer; s, suspensor; sc, seed coat. Scale bars: **a-l** = 20 μm

**Fig. 5:**
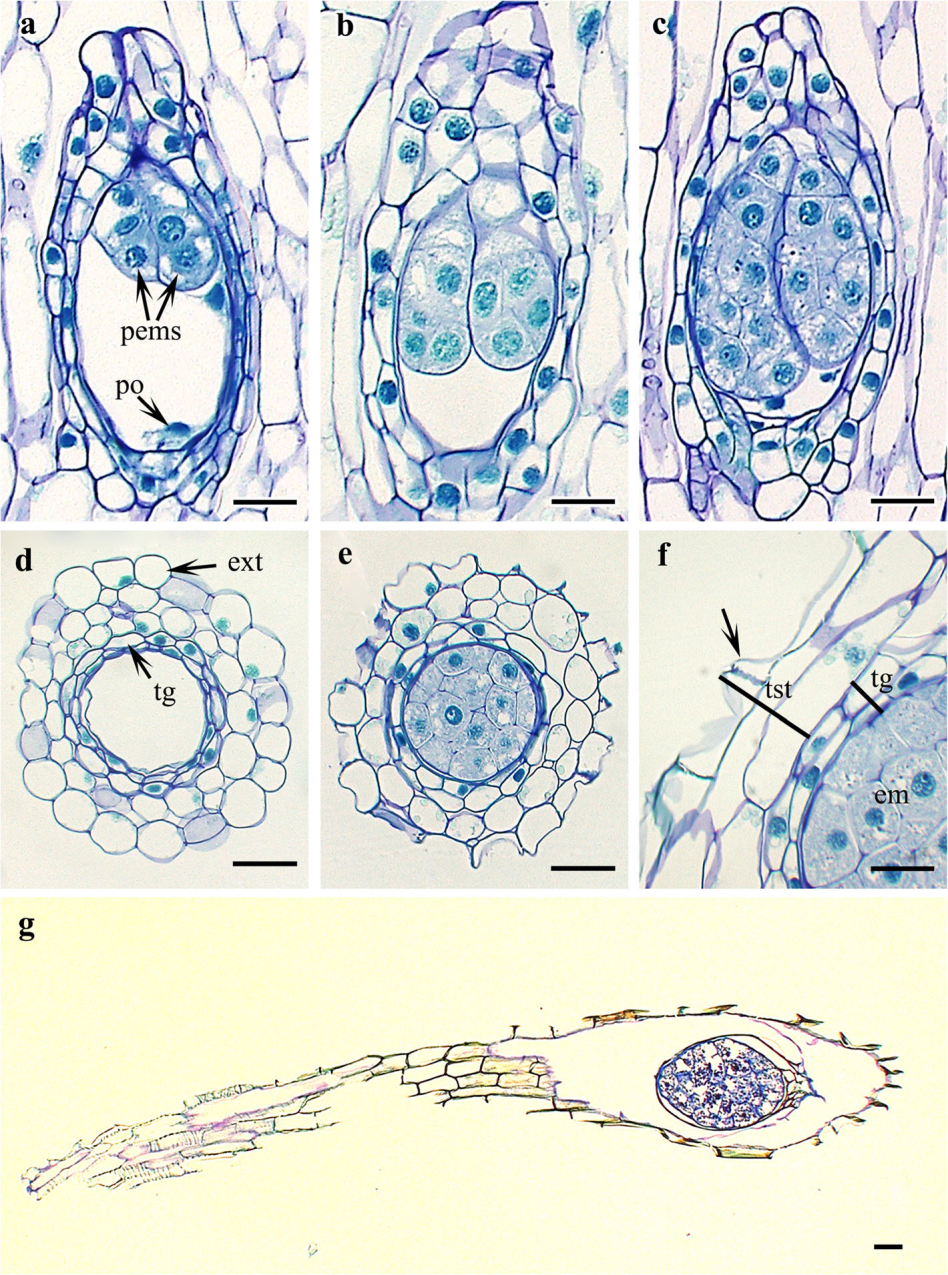
Polyembryony and seed coat development in *Cypripedium japonicum*. **a**–**c**. Polyembryony in different stages. **d**-**f**. Transverse division of seed showing seed coat development. **g**. Longitudinal section of mature seed. *Abbreviations*: em, embryo; ext., exotesta; pems, proembryos; po, polar nuclei; tg, tegmen; tst, testa. Scale bars: **a-e**= 20 μm,  **f**= 10 μm, **g** = 30 μm

The zygote, the precursor of embryo formation, is globular (Fig. 4a). The first division of the zygote is always transverse, resulting in equal-sized micropylar and chalazal cells. Mitotic division is immediately followed by rapid elongation of the micropylar cells, which acquire a large vacuole; in contrast, chalazal cells remain unchanged, with a dense cytoplasm (Fig. 4b). At the same time, division in the endosperm zygote also occurs, and the two-nucleate endosperm exists until the embryo reaches the globular stage (Fig. 4b, d, h, i). Both cells of the embryo divide transversely, producing a four-celled linear proembryo (Fig. 4c, d). No further division was observed in the two micropylar cells, which directly function as the suspensor, whereas the two chalazal cells, after a series of divisions, produce the whole embryonic body. Out of the two chalazal cells, transverse division occurs in the lower cell and longitudinal division occurs in the upper cell, and these four eventually give rise to the embryo proper (Fig. 4e, f). The series of divisions on both tiers of the cells of the embryo proper results in a 10-celled and then a 16-celled proembryo (Fig. 4g, h, i). Periclinal divisions from the outer layer of the 16-celled pro-embryo give rise to the protoderm (Fig. 4j, k, l). The suspensor was inconspicuous and represented by two cells. Normal mitotic division occurs in the suspensor, initially forming two cells that lack any special modification or development and that directly function as a suspensor. Polyembryony was also observed, although its frequency was quite low (Fig. 5a–c).

### Seed coat

The capsule of *C. japonicum* is elongated and fusiform to spindle-like in shape. Seeds are dispersed from longitudinal slits in the dry and ripened fruit in early September. The mature seeds are brown, minute, narrowly elliptical, and curved. The size range is 2.5 ± 0.3 mm in length by 0.15 ± 0.07 mm in width (measurements based on the seeds from 15 capsules of three consecutive years, five capsules each year, and at least 100 seeds from each capsule). Prior to maturity, the seed coat has four or five cell layers. The testa comprises two or three cell layers, and the tegmen is composed of two layers. The outermost exotestal cells are comparatively large and rounded to oval or irregularly shaped in cross-section. The exotesta is followed by a mesotestal layer and then an endotestal layer. Both of these layers are composed of similar types of cells as the exotesta but are smaller in size. The two innermost layers represent the tegmen, cells of which are smaller and elongated in cross-section (Fig. 5d–g). During maturity, the exotesta becomes papillate, the remaining layers degenerate, and the entire seed coat is represented by thin, papery layers.

### Reproductive calendar

**Fig. 6:**
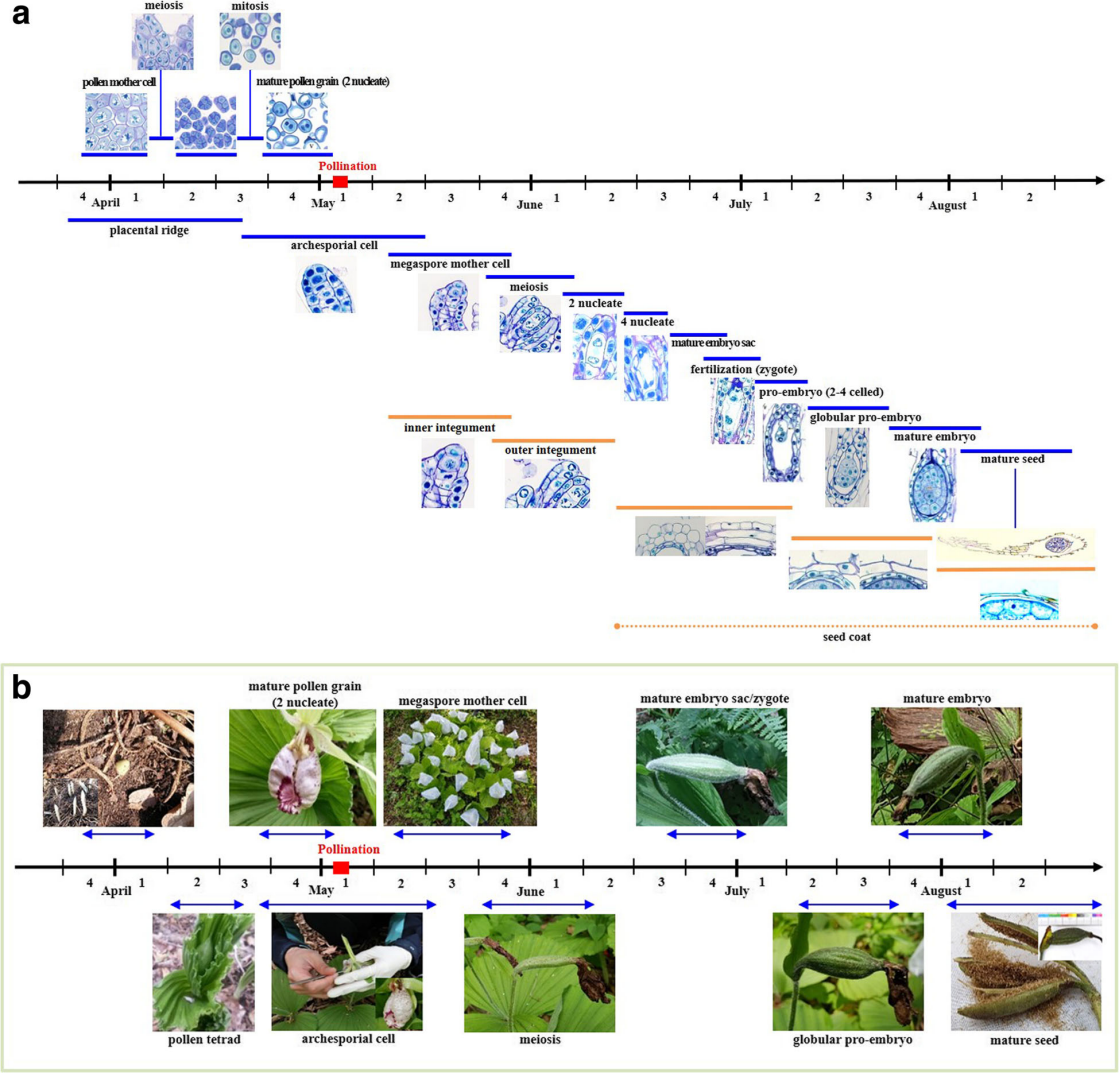
**a** Reproductive calendar of *Cypripedium japonicum* representing respective developmental stages. Coloured lines indicate the duration for the corresponding stage. Numbers (1–4) indicating weeks of the corresponding month. **b** Reproductive calendar of *Cypripedium japonicum* with flowers and fruit of main developmental stages. Blue lines indicate the duration for the corresponding stage. Numbers (1–4) indicating weeks of the corresponding month

The reproductive cycle of *C. japonicum* begins under the soil. Based on the observations presented in this study, a reproductive calendar of *C. japonicum* was prepared. All developmental stages over time are provided in Fig. 6a, b and supplementary file S1. Anther development starts in mid-March, when the whole plant is underneath the soil surface. The primary sporogenous cells directly function as pollen mother cells, and this process occurs from late March until early April. Meiosis in pollen mother cells results in variously shaped pollen tetrads by the middle of April. The nuclei of pollen grains divide by mitotic division during late April, and pollen grains are ready for pollination by early May. In our study population, the optimum period for pollination was May 2–7. Ovular development started approximately 2 weeks before pollination. The archesporial cell appeared during the third week of April and lasted until approximately the middle of May. The MMC was observed at 5–7 days after pollination (DAP) and lasted until the last week of May. Meiosis in the MMC started 2 weeks after pollination (WAP), and degeneration of the micropylar cell, followed shortly by the first nuclear division, occurred almost 4 weeks after pollination. The second and third nuclear divisions occurred 36–45 DAP. Thus, in *C. japonicum,* the female gametophyte matures 6–7 WAP. Fertilization was observed from late June to early July, approximately 7–8 WAP or 45–55 DAP, whereas the zygote started to divide by early July, and a two-to-four-celled linear proembryo was formed approximately 55–60 DAP. The constant cell division in the four-celled proembryo resulted in an eight- and then 16-celled globular embryo approximately 65 DAP. Within 72–80 DAP, the whole interior of the seed was filled with embryo. Mature seeds were harvested 90 DAP.

## Discussion

This study provides a complete overview of the embryology of the endangered slipper orchid *C. japonicum*. In nature, this nonrewarding orchid reproduces by both sexual and vegetative methods. However, the general occurrence of fruit set in nonrewarding orchids, including *C. japonicum*, under natural conditions is very low [28, 41]. It is quite understandable that such nonrewarding species are often challenged by pollinator limitation and thus exhibit low reproductive success in nature [29, 41, 42]. Within the genus, some endangered species, such as *C.* *fargesii * and *C. macranthum,* exhibit extremely low conversion rates of flowers to fruit [43], whereas *C. calceolus*, *C. montanum*, and *C. parviflorum,* which are pollinated by a wide variety of bees, show fruit set percentages as high as 45–85% [19]. However, Edens-Meier et al. [18] and Bernhards and Eden-Meier [14] believed that beyond pollinator limitation, there must be other genetic-based and/or environmental stress preventing fruit or seed maturation following successful pollination. Suetsugu and Fukishima [28] found that the fruit set percentage of *C. japonicum* by natural pollination was only 14.9%, and the value was even lower (i.e., only 5.2–7.7%) in the samples studied by Sun et al. [29]. Although we carried out hand pollination in our study population, we observed a very low fruit set percentage (≤5%) in naturally pollinated populations near our sampling site, which is almost consistent with the results of Sun et al. [29]. The fruit set percentage in hand-pollinated populations in our experimental site was much higher (60–65%), more than 12-fold that in the naturally pollinated population. Hand-pollination experiments have already confirmed self-compatibility in *C. japonicum* [28, 29]; however, we performed cross-pollination between individuals of both the same and different clumps of the population.

### Microsporogenesis and microgametogenesis in *Cypripedium* versus other Orchidaceae

A majority of recent embryological studies of the family Orchidaceae have focused on ovule, embryo, and suspensor development. Pollinia and male gametophyte developmental studies for orchids have rarely been conducted, and only a few studies are available on *Spiranthes* and *Ophrys* species [39, 44, 45]. Sood and Rao [31] had previously studied the embryology of *C. cordigerum*. This study attempted to provide a comprehensive statement regarding the embryology, including anther structure and male gametophyte development, of *C. japonicum*. In *C. japonicum,* anther development occurs very early (possibly in mid-March), even when the entire plant remains underneath the frozen soil surface. In previous studies, the monocotyledon type of anther wall formation has been observed in some orchid species [46–48]; however, due to a lack of anthers in the early developmental stages, wall development in *C. japonicum* could not be observed here. Nevertheless, the anther wall, from the beginning of microspore mother cell development to the end of microsporogenesis, was six- or seven-cell layered. This is consistent with the findings of Sood and Rao [31], who mentioned six to eight layers in *C. cordigerum*, although Wirth and Withner [7] reported a five-cell-layered anther wall in most terrestrial orchids. In contrast, Aybeke [44] found only four-layered anther walls in *Ophrys mammosa*. In this study, we found a single-layered tapetum, except at locations where a few cells divided periclinally, forming two layers. A partly two-layered tapetum in the area between the two microsporangia has been described for *Ophrys mammosa* [44]. In contrast, Sood and Rao [31] described a two- or three-layered tapetum in *C. cordigerum* that is infrequent for most orchids [33]. More interestingly, Sood and Rao [31] revealed a two-layered endothecium in the mature anther wall of *C. cordigerum*, formed by the endothecium and the outermost middle layer. However, in *C. japonicum,* only the endothecial layer develops such thickening, and none of the remaining middle layers persist in the mature anther. Moreover, none of the previous works have mentioned a double-layered endothecium in the family [7, 32, 33, 44, 47–50]. This disparity in the nature and number of wall layers makes this feature a worthwhile taxonomic and embryological development criterion in Orchidaceae [3, 44, 51].

Early studies suggested that a simultaneous type of cytokinesis in microspores is the characteristic feature of the family Orchidaceae [3, 33]); however, successive types are also often observed in this family [39, 44]. In this study, we found a simultaneous type of microsporogenesis in *C. japonica*. This is consistent with the findings of Sood and Rao [31] and Poddubnaya-Arnoldi [36], who described simultaneous microspore development in other *Cypripedium* species, although Guignard [52] previously reported the successive type for this genus. This variation in cytokinesis in *Cypripedium* species might not be surprising, as both types of cytokinesis have been observed in *Spiranthes sinensis* [34, 45] and in a few other angiosperms, such as *Rauvolfia canescens* [53], *Rauvolfia serpentina* [54], and *Catharanthus pusillus* [55]. We observed that the resultant pollen tetrads are isobilateral, tetrahedral, linear, or T-shaped, which are common tetrad types in the family, although only decussate, isobilateral, and tetrahedral types have been observed in *C. cordigerum* [31]. The mature pollen grains are exclusively single and two-celled in *C. japonicum*, as observed in other *Cypripedium* spp. [31, 36].

### Megasporogenesis and megagametogenesis in *Cypripedium* versus other Orchidaceae

Ovule, embryo sac, and megagametophyte development in *C. japonicum* follow the usual pattern described in other Orchidaceae. During this time, two dermal cells of the nucellar filament just below the MMC become enlarged and are divided by a transverse wall. Thus, both integuments are dermal in origin, as described for *C. cordigerum* [31], and the inner integument is usually involved in micropyle formation. Strangely, Poddubnaya-Arnoldi [36] reported a unitegmic ovule in *Cypripedium insigne* that is not common in the genus, even in the family Orchidaceae [33]. Initially, the division of MMCs usually results in an equal-sized dyad; however, in this species, only the chalazal dyad proliferates and becomes functional, which results in the development of the whole embryo sac, while the micropylar cell soon degenerates. Pace [30], who observed fertilization and embryogenesis in four *Cypripedium* species, described the four-nucleate bisporic embryo sac as characteristic of the genus *Cypripedium* and thus termed it the ‘*Cypripedium* type’ of embryo sac. However, this was later corrected by other researchers who found five- to eight-nucleate bisporic embryo sacs instead of four-nucleate ones in different *Cypripedium* species [31, 35, 36, 55–59]. The results of this study are relatively similar to the latter findings, as we observed a six-nucleate bisporic mature embryo sac. In this case, the antipodal is represented by a single cell because one nucleus from each side moves to the center of the embryo sac and forms a binucleate central cell that produces the endosperm zygote after triple fusion. This failure of subsequent nuclear division in chalazal nuclei is termed the ‘strike’ phenomenon. Although this characteristic has been observed in both monosporic and bisporic orchids, the number of nuclei in the megagametophyte and the nature of antipodal cells are apparently independent features in various genera of the family. In some monosporic orchids, the failure of consecutive mitosis in chalazal cells ultimately results in the absence of antipodal cells in the mature embryo sac [60, 61]. On the other hand, in bisporic orchids such as *Paphiopedilum* spp., one of the micropylar nuclei and two chalazal nuclei of the four-nucleate-stage embryo sac fail to divide, and thus, the mature embryo sac comprises five nuclei [62].

### Pollination and fertilization in *Cypripedium* versus other Orchidaceae

The pollen tube enters through the micropyle, and fertilization likely occurs between 7 and 8 WAP. Despite the delayed fertilization process, the growth of pollen tubes in the style does not occur at the same rate in *C. japonicum* as the growth of the mass of pollen tubes seen near the placental cells at 21 days after pollination. This period is likely consistent with that observed by Duarte et al. [61], who found spreading pollen tubes on placental lobes at 20–25 DAP in *Acianthera johannensis*. A previous study on *Cypripedium* suggested that the delivery of pollen tubes to ovules is not a synchronous process but occurs continuously over a series of days after 15 DAP [18], and the results of our study are consistent with this finding. On the other hand, this extended period between pollination and fertilization is common in Orchidaceae and was even independently evolved by many orders of eudicotyledons [18, 37, 61, 63, 64]. There are two types of interpretations for this delayed fertilization: Sogo and Tobe [64] described incomplete megasporogenesis at the time of pollination, whereas Writh and Wrthner [7] and Arditti [65] confirmed late ovule development within Orchidaceae. Based on our results, late megasporogenesis is probably the correct interpretation for this delayed fertilization in *Cypripedium* [18]. We confirmed that double fertilization and triple fusion evidently occur in *C. japonicum*, although the primary endosperm is binucleate and short-lived. The degeneration of endosperm nuclei occurs either before or after the embryo has reached the 16-cell stage. Previously, Pace [30] explained the common occurrence of two (or, rarely, four) endosperm nuclei in *Cypripedium* spp., but no triple fusion was properly observed. According to Pace [30], the primary endosperm is formed by the fusion of the polar nucleus, one synergid, and one male nucleus, but on the basis of this study, we reject this possibility for *C. japonicum*. Later, Sood and Rao [31] also found a binucleate primary endosperm in *C. cordigerum,* whereas Poddubnaya-Arnoldi [36] recorded a six-nucleate endosperm for *C. insigne*. The maximum number of endosperm nuclei recorded in the family is 12 in *Vanilla* and 16 in *Galeola septentrionalis* [66]. Moreover, some previous studies disputed double fertilization in orchids. For instance, according to Savina [67], in *Listera ovata* and *Ophrys insectifera*, only one male gamete fuses with the egg, but a second male gamete does not fuse with the secondary nucleus; thus, no triple fusion occurs. Correspondingly, Maheshwari and Narayanaswami [68] reported that only one male gamete is released from the pollen tube in *Spiranthes australis*; thus, fertilization of polar nuclei is not likely to occur in that species. Tarasaka et al. [69] suggested the failure of generative cell division in pollen, resulting in only one sperm cell in *S. sinensis* and thus in no male gamete remaining for triple fusion; however, this was later disputed by Battaglia [70]. Recently, by in vitro pollen germination of *S. sinensis*, Wang et al. [39] found two male gametes in the pollen tube and suggested that triple fusion in this species is expected.

### Comparative embryogenesis in *Cypripedium* versus other Orchidaceae

One of the most notable features during the course of embryo development in *Cypripedium* is the lack of an elaborated suspensor cell. The suspensor is likely to be two-celled in *C. japonicum*. Previous studies suggested that the suspensor in *Cypripedium* is either totally absent [71, 72] or represented by one or two inconspicuous cells [30, 31, 58, 71]. It is interesting that a majority of structural studies of embryo development in Orchidaceae have focused on suspensor development; even Swamy’s [4] embryo classification idea was based on the suspensor structure. According to Swamy’s five embryo categories based on suspensor morphology in Orchidaceae, *Cypripedium* belongs to the type I category, in which the initial suspensor cell does not undergo any division but remains without much elongation. Similar to the findings in *Cypripedium* species, Prakash and Lee [46] and Tohda [73] reported unicellular and vestigial suspensors in *Spathoglottis plicata* and *Lecanorchis* spp., respectively. Moreover, in *Hetaeria shikokiana,* a unicellular suspensor develops into a long projection through the micropyle, becomes twisted, and enters the tissue of the placenta [74]. The function of the unique vacuolated suspensor of the orchid remains uncertain, although Lee et al. [2] found specific structural specialization with possible ‘transfer cell’ morphology in *Paphiopedilum delenatii*. However, this is as yet unclear for taxa such as *Cypripedium* in which a specialized suspensor is lacking. Thus, to understand the definite structural specialization of suspensors in such species, further studies focusing on suspensor ultrastructure are required.

The first division of the zygote in *C. japonicum* follows a regular pattern, forming a terminal cell and a basal cell. Swamy [4] classified orchid embryos into three types based on the embryonic cell division pattern. According to Swamy’s report, *Cypripedium* belongs to group A based on the cell division pattern of the developing embryo and type I based on suspensor morphology. However, Sood and Rao [31] categorized the embryogeny of *C. cordigerum* under group B of Swamy’s [4] classification, stating a greater share for the basal cell in the organization of the mature embryo. Before Swamy’s classification, Carlson [57] reported a one-celled suspensor in *C. parviflorum* originating from the basal cell and the entire embryo developing from the terminal cell alone. The results of our study were fundamentally consistent with those of Carlson [57] rather than those of Swamy [4] and Sood and Rao [31], as the basal cell does not take part in the formation of embryonal mass in *C. japonicum*. One of the most notable findings in *C. japonicum* is that the second and third divisions in the terminal cell also occur by transverse wall formation, resulting in a five-celled linear proembryo. Longitudinal division occurs in the terminal cell of the five-celled proembryo, forming two juxtaposed cells. Meanwhile, the micropylar cell divides transversely, forming a two-celled suspensor. Based on the results of this study, we confirm that *C. japonicum* does not follow the pattern of embryo and suspensor development described earlier for this genus.

### Conservation perspective

Low fruit set rates and irregular germination in nature as well as under in vitro conditions are major bottlenecks for the conservation of rare and threatened orchids [75]. One of the major threats to decreasing populations of *C. japonicum* is the complex pollination mechanism with low fruit set rates and irregular germination under natural conditions. Although in vitro seed germination has received the most attention for different *Cypripedium* species, no successful in vitro germination methods have been suggested for *C. japonicum*. Several reports have suggested that immature seeds exhibit better in vitro germination than mature seeds in *Cypripedium* species [76, 77]. According to de Pauw and Remphrey [76], seeds collected at 8 WAP exhibited a sharply decreased germination percentage in three *Cypripedium* species. Recently, Jiang et al. [77] found the optimum germination percentage in *C. lentiginosum* to be from seeds collected at 90–105 DAP, at which time the embryo is in the early globular to globular stage. In *C. japonicum,* the embryo in the early globular to globular stage was observed at 9–10 WAP or 65–75 DAP. Thus, the seeds collected near this period could be useful for in vitro seed germination for this species. It is hoped that the reproductive cycle presented in this study will be useful in future studies for selecting the appropriate embryonic stage for germination experiments.

## Conclusions

This study presents comprehensive embryological features of the endangered orchid *C. japonicum* and provides a reproductive calendar for the species. Although some previous investigations have reported a number of embryological features for the genus *Cypripedium*, the data pertaining to *C. japonicum* in this study are entirely new. Our overall comparison of gametophyte and embryo development features suggests that previous reports of *Cypripedium* embryology are not sufficient for characterization of the entire genus. Moreover, some of the previously reported features are ambiguous and thus need to be corrected by new research. This study helps to clarify some contradictory findings from previous reports. Given these aspects, the results of this study will certainly be helpful for future research and will provide a fundamental reference for the embryological data for the genus *Cypripedium* and likewise for the family Orchidaceae.

## Methods

### Plant materials

*Cypripedium japonicum* is a 20–40 cm tall terrestrial orchid that grows under both mature and successional deciduous forests on hillsides in China, Korea and Japan [78]. The species is also found in the understory of bamboo forests in lowlands in Japan [79]. Several clumps of *C. japonicum* were grown inside a fence that was built and protected by Korea National Arboretum near one of the six natural populations that grows in Korea (see Chung et al. [80]). Formal identification of the plant was carried out by a group of plant taxonomists, including Dr. Sungwon Son (one of the authors) in Korea National Arboretum.

### Hand pollination

*Cypripedium japonicum* is a nonrewarding orchid, and the reproductive success rate is obviously relatively low for such plants [81, 82]. Sun et al. [29] suspect infrequent pollination to be the reason for low fruit production in this species. Therefore, to ensure adequate samples for experiments with a high fruit set rate, flowers were manually cross-pollinated by transferring pollen of one flower onto the stigma of another flower, with an effort not to limit cross-pollination to the same clump. Altogether, 130–170 flowering plants during three consecutive years were counted and hand pollinated in all clumps. A pollinium was detached from a flower with a pointed pin set, rinsed with 70% ethanol prior to use, and attached to the stigma of another flower. To avoid unwanted pollination by insects, the pollinated flowers were covered by a finely netted nylon bag.

### Sampling

The plant materials were collected during the months of March to September for three consecutive years (2016–2018) with the permission of Korea National Arboretum, and the samples remaining after the experiment and all the observed slides were preserved in the seed testing laboratory. Specimens and seed samples (voucher no. KNASB 17,562–17,573) were deposited in the seed bank of Korea National Arboretum, Korea. For a comprehensive study of embryology, developing flowers and fruits were collected at a regular time, every week, before and after pollination. At least three to five samples representing all developmental stages (before and after pollination) were collected every week. Sample collection was started at the end of March (digging the underground flower buds), when new shoots start to appear, until late September, when the fruit is completely ripened and the fruit wall starts to rupture for seed dispersal. Details regarding the time (in weeks) and the corresponding developmental stages are provided in supplementary file S1. In the early collections, particularly those from March, which were acquired mainly to study male gametophyte development, all the developing shoots were underneath the soil surface. All the collected samples were fixed in formalin/acetic acid/50% ethyl alcohol (FAA) at a ratio of 90:5:5 per 100 ml for at least 3–5 days and preserved in 50% ethyl alcohol [83].

### Light microscopy

Preserved flower buds, open flowers, and fruit were dehydrated in an ethyl alcohol series (50, 70, 80, 90, 95, and 100%) for at least 24 h in each stage. After complete dehydration, the samples were passed through infiltration solutions of alcohol/Technovit (3:1, 1:1, 1:3, and 100% Technovit) and then embedded in polymerization solution prepared by mixing Technovit 7100 resin stock solution with Technovit 7100 harder II. Histological blocks were prepared from each embedded material with Histoblock and Technovit 3040 so that the mold could be removed and the material sectioned. Serial sections of 4–6 μm thickness were cut using a Leica RM2255 rotary microtome (Leica Microsystems GmbH, Germany) with disposable blades, stuck onto histological slides and dried using an electric slide warmer for 12 h. Staining was performed with 0.1% toluidine blue O for 60–90 s. Following staining, the slides were rinsed in tap water and again dried with an electric slide warmer for more than 6 h to remove water [84]. Entellan (Merck Co., Germany) was used for mounting, and mounted slides were pressed using metal blocks for a couple of days to remove air bubbles. These permanent slides were observed under an AXIO Imager A1 light microscope (Carl Zeiss, Germany), and photomicrographs were taken with an attached camera system in the microscope. For the record of all developmental stages of sporogenesis and gametogenesis, a minimum of five samples of each stage were sectioned and observed.

## Supplementary information

**Additional file 1 **Sampling time with respective developmental stages of *Cypripedium japonicum. (XLSX 10 kb)*

## Data Availability

The datasets used and/or analysed during the current study are available from the corresponding author on reasonable request.
